# Dupilumab as a rescue therapy for steroid-dependent eosinophilic gastritis in a child unresponsive to elimination diet: a case report

**DOI:** 10.3389/fped.2026.1755630

**Published:** 2026-04-13

**Authors:** Marisa Piccirillo, Giovanni Di Nardo, Emanuela Pilozzi, Silvia Furio, Vincenza Patrizia Di Marino, Valentina Pucinischi, Emanuele Dilaghi, Pasquale Parisi, Alessandro Ferretti, Maurizio Mennini

**Affiliations:** 1 Department of Neurosciences, Pediatric Unit, Mental Health and Sensory Organs (NESMOS), Faculty of Medicine and Psychology, Sapienza University of Rome, Sant'Andrea University Hospital, Rome, Italy; 2 Department of Pediatric Specialties, Pediatric Gastroenterology and Endoscopy Unit, Santobono Pausilipon Children's Hospital, Naples, Italy; 3 Department of Clinical and Molecular Medicine, UOC Anatomia Patologica, Sant' Andrea Hospital, University “La Sapienza”, Rome, Italy; 4 Department of Medical-Surgical Sciences and Translational Medicine, Sant'Andrea Hospital, Sapienza University of Rome, Rome, Italy

**Keywords:** dupilumab, eosinophilic esophagitis, eosinophilic gastritis, monoclonal antibody therapy, pediatric gastrointestinal disorders

## Abstract

**Background:**

Eosinophilic gastrointestinal diseases (EGIDs) are chronic, rare and heterogeneous disorders characterized by eosinophilic infiltration of gastrointestinal tract, resulting in gastrointestinal dysfunction. Although standard-of-care therapeutic strategies for eosinophilic esophagitis (EoE) are well-defined, limited evidence on successful treatment of eosinophilic gastrointestinal disorders beyond eosinophilic esophagitis (non-EoE EGIDs) in children is available. While dupilumab, a monoclonal antibody that inhibits interleukin (IL)-4 and IL-13 signaling, is currently approved in children with EoE, only anecdotal studies assessed its effects in children with non-EoE-EGIDs.

**Case presentation:**

This case report describes the successful use of dupilumab as a rescue therapy for a 14-year-old male with concurrent EoE and Eosinophilic Gastritis (EoG), unresponsive to dietetic approach. Despite initial responsiveness to steroid treatments, the patient experienced challenges in tapering off oral steroids without relapse. The administration of dupilumab at a dosage of 300 mg once a week led to clinical, endoscopic, and histological improvement after three months of therapy, highlighting its efficacy as a well-tolerated treatment option.

**Conclusions:**

The successful use of dupilumab as a single-therapy therapy in a steroid-dependent child, who did not respond to dietary interventions, highlights its potential in mitigating the development of steroid-related side effects. This report underscores the need for further research and clinical trials to deepen our understanding and optimize the use of dupilumab and other monoclonal antibodies in managing pediatric EGIDs, establishing a basis for the future development of treatment guidelines and protocols.

## Introduction

Eosinophilic esophagitis (EoE) is a chronic allergen/immune-mediated disease characterized by symptoms of esophageal dysfunction and eosinophilic infiltration of the esophageal mucosa (≥15 eosinophils/high-power field) in the absence of secondary causes of eosinophilia (1, 2). EoE is a multifactorial disease in which Th2 inflammatory pathways and the consequent production of Th2 cytokines (IL-4, IL-5, IL-9 and IL-13) play a pivotal role (3, 4). Thus, the US food and drug administration in 2022 approved dupilumab for the treatment of EoE in patients older than 1 year (5). Dupilumab is a human monoclonal antibody binding to the *α* subunit of the IL-4 receptor, thus inhibiting either interleukin-4 (IL-4) and interleukin-13 (IL-13) (6, 7). It can be administered by subcutaneous injection and is currently approved in children for the treatment of asthma, atopic dermatitis and eosinophilic esophagitis (7). According to the recent ESPGHAN/NASPGHAN guidelines, Eosinophilic Gastritis (EoG) is recognized as a component of eosinophilic gastrointestinal disorders beyond eosinophilic esophagitis (non-EoE EGIDs). These are rare chronic immune-mediated disorders of the gastrointestinal (GI) tract characterized by eosinophilic inflammation of the mucosa, which can lead to organ dysfunction (8).

EoG appears to be driven by a similar Th2 mechanism as EoE, evidenced by elevated levels of IL -4, IL -5, and IL -13 (9, 10). Current guidelines recommend oral systemic or topical steroids, elimination diets (particularly cow's milk), proton pump inhibitors (PPI) for managing EoG (8). However, there remains a scarcity of data regarding standard-of-care treatment for non-EoE EGIDs. In this context, we present our experience with the use of dupilumab as a rescue therapy for concurrent EoE and EoG.

## Case presentation

Upon presentation, our patient, a 10-year-old male weighing 40 kg, reported heartburn, regurgitation, and vomiting occurring up to twice a week. He had no history of non-steroidal anti-inflammatory drugs (NSAIDs) intake, atopic dermatitis or allergic rhinitis, although his family history was positive for atopic condition (allergic rhinitis and drug allergy). Laboratory tests revealed no signs of anemia or eosinophilia (hemoglobin 11.1 g/dL, eosinophils 230/mm3). Despite undergoing a 2-month trial therapy with proton pump inhibitors (lansoprazole, 30 mg/day), his symptoms did not improve.

Consequently, an esophagogastroduodenoscopy (EGD) was performed, during which two biopsies were obtained from the distal and proximal esophagus, gastric body, fundus, antrum, and duodenum.

**Figure 1:**
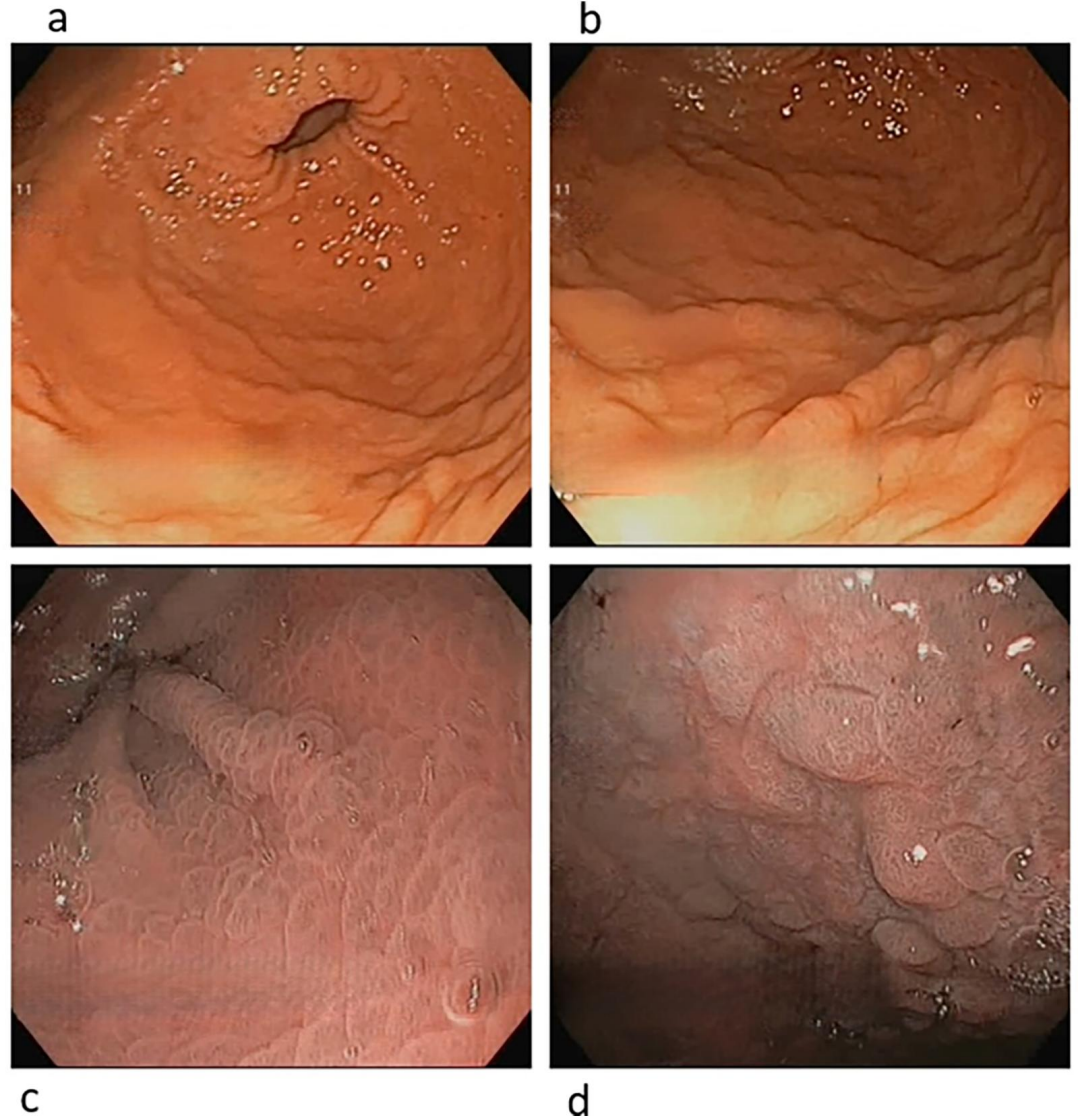
Endoscopic appearance of gastric mucosa at the baseline upper gastrointestinal evaluation. At the white light evaluation, gastric mucosa was diffusely erythematous, and a mild nodular appearance of the mucosa was noticed, more evident in the antrum **(a)** than in the gastric corpus **(b)** At the electronic chromoendoscopy (Narrow Band Imaging) evaluation, the mucosa did not show the presence of intestinal metaplasia and no gastric area showing irregular mucosal and/or vascular pattern was detected, both in the antrum **(c)** and in the corpus **(d)**.

**Figure 2:**
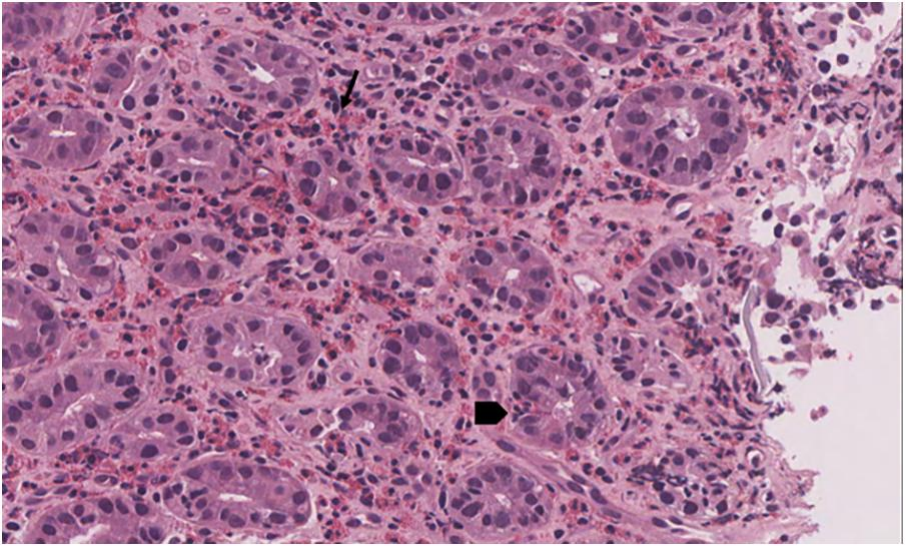
Gastric biopsy showing prominent eosinophilic infiltrate in lamina propria (arrow) and intraepithelial (arrow head) [hematoxylin-eosin 40x].

**Figure 3:**
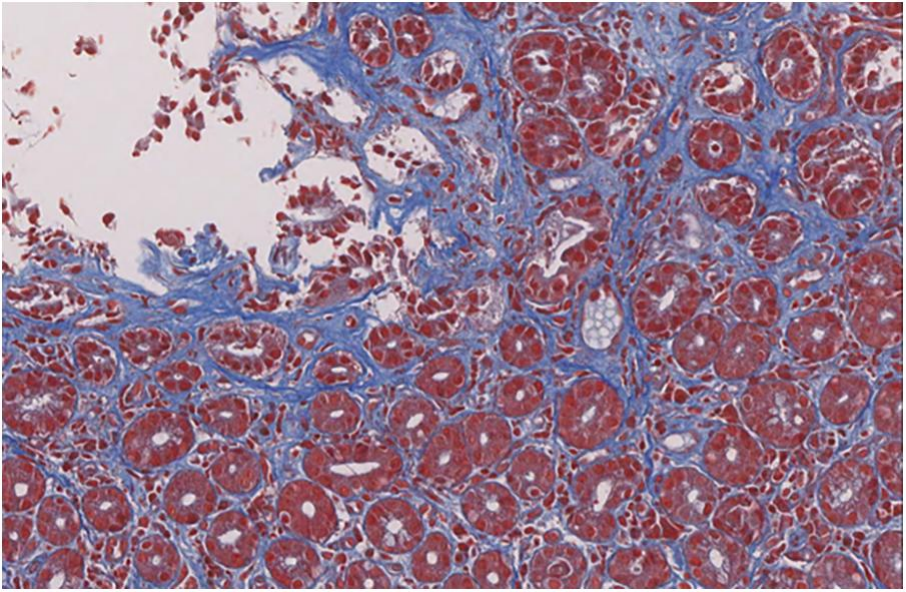
Trichrome staining shows fibrosis of superficial lamina propria [trichrome staining 30x].

Gastric mucosa appeared erythematous and edematous, with microulcerations in the antrum (Figure 1). Biopsies revealed EoG, characterized by dense eosinophil infiltration of the lamina propria (>100 eos/HPF), eosinophil microaggregates, and severe fibrosis in the lamina propria (Figures 2, 3). Eosinophilic count in esophagus and duodenum did not reveal EoE or eosinophilic duodenitis. *Helicobacter pylori* was not found in the mucosa sample, and stool culture and parasite tests were negative.

Initiating an empiric elimination diet (milk, egg and soy) did not yield significant clinical improvement. After 3 months since the beginning of the food elimination diet, EGD and colonoscopy were both performed. The only macroscopic anomaly detected during the endoscopic procedure was the nodular, erythematous and edematous appearance of gastric mucosa. Biopsies collected from all the segments explored showed persistent EoG (>100 eos/HPF, lamina propria fibrosis) and, in addition, EoE characterized by eosinophil infiltration (70 eos/HPF) and eosinophil aggregates in esophageal mucosa. Random biopsies obtained from the colon revealed no histopathological abnormalities. Fecal calprotectin was within normal limits (20 mcg/g). The patient was referred to the allergy unit, where allergy tests were conducted. Specific Immunoglobulin E (IgE) levels for milk, soy, wheat, egg-white, and yolk were normal. Similarly, the skin prick test (SPT) for cow's milk protein, egg, corn, codfish, soy, wheat, rice, peanut, hazelnut, walnut, tomato, and apple returned negative results.

Taking into account the patient's dietary history, which revealed a significant consumption of carbohydrates, particularly pasta and bread, we decided to initiate a four-food elimination diet targeting milk, egg, soy, and wheat.

After 6 months since the beginning of the elimination diet, a clinical and endoscopic remission of both EoE and EoG was observed, consistent with a histologic remission of EoE (10 eos/HPF) but not with a histologic remission of EoG, since biopsy revealed a persistent eosinophilic infiltration of the gastric mucosa (>100 eos/HPF, lamina propria fibrosis). As a result, the previously eliminated four foods were successfully reintroduced into the patient's diet, and locally acting corticosteroid therapy (enteric-coated budesonide capsule 9 mg, 0.2 mg/kg/day administered after opening the capsule) was initiated.

While on steroid therapy, symptoms subsided, and complete endoscopic and histological remission (Supplementary Material 1, 2) was observed both in esophagus and in stomach (1-2 eos/HPF and 8 eos/HPF respectively) after 6 months since the beginning of budesonide.

**Figure 4:**
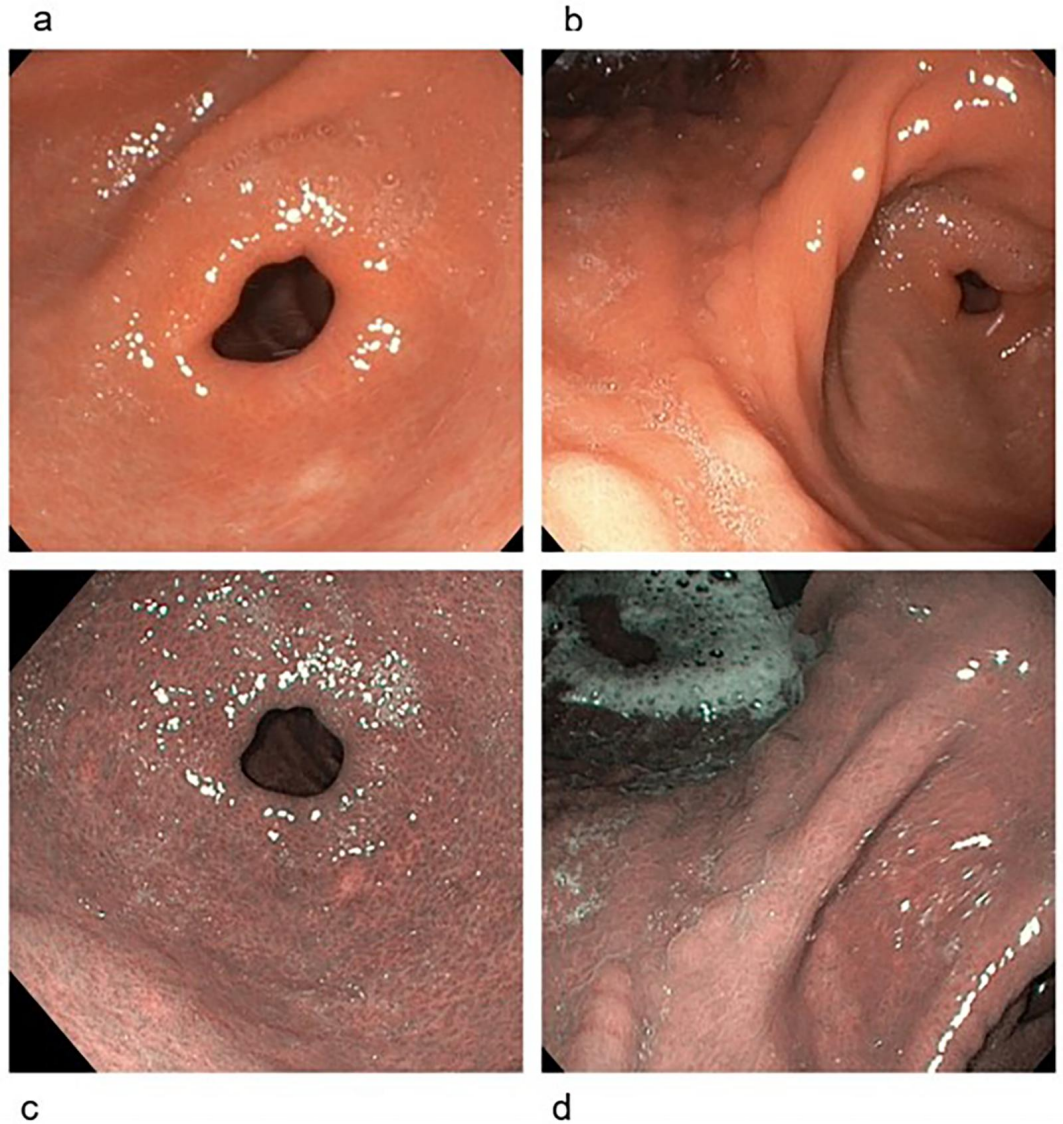
Endoscopic appearance of gastric mucosa after dupilumab therapy. At the white light evaluation, gastric mucosa was slightly erythematous, and a minimum nodularity of the mucosa was noticed, both in the antrum **(b)** and in the corpus **(a)**. At the electronic chromoendoscopy (Narrow Band Imaging) evaluation, the mucosa did not show the presence of intestinal metaplasia and no gastric area showing irregular mucosal and/or vascular pattern was detected, both in the antrum **(c)** and in the corpus **(d)**.

However, upon attempting to taper off budesonide at a dosage of 3 mg/die, a histological relapse of EoG (40 eos/HPF) and EoE (60 eos/HPF) was observed (Supplementary Material 3). Considering the steroid-dependent EoG and the additional EoE, in agreement with the patient's parents, we opted for dupilumab as an off-label therapy administered by subcutaneous injection at a dosage of 300 mg once a week, discontinuing oral budesonide. At that time, the patient was 14 years old and weighed 50.5 kg. After 3 months of therapy and 12 doses of dupilumab, the patient finally achieved clinical remission and EGD partially improved, revealing only slight erythematous and edematous gastric mucosa, with minimum nodularity (Figure 4). Biopsies collected from esophageal mucosa revealed only mild spongiosis, in absence of eosinophilic infiltrates, while histologic examination of biopsies collected from gastric mucosa showed mild inflammatory infiltrate limited to *lamina propria* (40 eos/HPF), without fibrosis (Supplementary Material 4). Moreover, the patient did not report vomiting nor pyrosis, while significant improvement in quality of life during subsequent follow-up visits.

Six months after initiating dupilumab, a clinical, endoscopic and histological remission of both EoE and EoG was achieved (4 eos/HPF and 10 eos/HPF respectively); this remission persisted one year after starting therapy.

## Discussion and conclusions

Currently, there is limited evidence on the use of dupilumab as a treatment for non-EoE EGIDs (11–14).

The aims of medical therapy in patients with EoE include histologic remission (eosinophil count of <15 eos/HPF), symptoms resolution, prevention of submucosal fibrosis and consequently remodelling, and improvement in children's Quality of Life (QoL) (15).

According to current guidelines, first-line non-surgical therapeutic options include food elimination diets, proton pump inhibitors (PPIs) and topical corticosteroids (16). These strategies have demonstrated success in achieving symptoms resolution and normalization of eosinophil counts in biopsies for children with EoE (17–19).

Given the emerging prevalence of non-EoE-EGID in the last decade, there is a rapidly growing interest in potential treatments (20). Current guidelines suggest a peak eosinophil count of ≥30 eos/HPF as the diagnostic threshold for EoG. Supportive histological findings include eosinophilic glandulitis, eosinophilic abscesses, muscularis mucosa infiltration, and lamina propria ﬁbroplasia or smooth muscle hyperplasia. Although universally accepted histologic response criteria are lacking, treatment goals for non-EoE EGIDs encompass resolution of symptoms, improving endoscopic and histological abnormalities achieving normal growth and development, and preventing disease complications (8). Current guidelines suggest the use of systemic (at appropriate doses followed by timely tapering) or topical steroids and food elimination diets, targeted onto the severity of patient's disease, to induce and maintain remission (8).

In non-EoE-EGID, therapy with oral prednisone at a dose of 0.5-1 mg/kg with a maximum dose of 40 mg for 2 weeks is recommended to induce remission, followed by tapering over 2-8 weeks (8, 21). As topical steroid therapy, enteric-coated budesonide capsules, normally used in ileo-colonic Crohn's disease, can be administered. The suggested dose of budesonide is 9 mg/day, reducible to 6 mg/day and consequently to 3 mg/day for maintenance therapy (8, 21, 22).

The emerging role of monoclonal antibodies in children with EoE has been extensively discussed.

Dupilumab, a human monoclonal antibody targeting the interleukin-4 receptor alpha (IL-4R*α*), inhibits the signaling of IL-4 and IL-13, crucial initiators of type 2 inflammation (6, 7). Recently approved in both the USA and Europe, dupilumab is indicated for children with EoE aged 1 year or older, weighing at least 40 kg, who are unable to tolerate conventional treatment or have experienced failure with conventional treatments (5, 7, 23, 24).

Previous literature exploring the use of dupilumab or other monoclonal antibodies as a treatment for children with non-EoE-EGIDs is limited. Patel et al. reported a case series involving three patients with non-EoE-EGID treated with dupilumab: patient 1 [affected by EoE, EoG, and Eosinophilic duodenitis (EoD)] and patient 2 [affected by EoE, EoG, EoD, and Eosinophilic jejunitis (EoJ)] were unresponsive to standard treatment; patient 3 (affected by EoE and EoJ) experienced side effects from corticosteroids. All patients concurrently suffered from an atopic condition (asthma, food allergies, or atopic dermatitis). In Patel's study, patients were treated with dupilumab subcutaneously, with a dosage of 200 mg for patient 1 and 300 mg for patients 2 and 3, administered every 2 weeks, resulting in clinical, endoscopic, and histological remission (11).

A Japanese study reported a case of a 15-year-old girl diagnosed with EoG, EoD, and Eosinophilic colitis (EoC) and asthma who developed food tolerance after starting dupilumab. However, this study did not report exact dosage of the drug administered nor histologic findings after clinical remission (12).

Moreover, Sia et al. described the effects of dupilumab on non-EoE-EGIDs in adult population: 2 patients had both EoG and EoD, 3 patients had EoG without EoD, and 7 patients had EoD without EoG. All patients suffered from concurrent EoE. Dupilumab 300 mg was administered every week for their co-occurring EoE, for a median of 36.9 weeks in EoG patients and 48.7 weeks in EoD patients. While on dupilumab, 2 patients (40%) with EoG and 3 patients (33.3%) with EoD had complete symptoms remission. Only 1 patient reported mild joint pain, a previously reported adverse event associated with dupilumab. Only 2 patients with EoD did not experience histologic remission but had improvements in symptoms (14).

Recently, Lionetti and colleagues presented a case of a 14-year-old boy affected by allergic rhinitis and EoE, EoG, eosinophilic enteritis (EoN) and EoC with oral steroid dependence, who started an off-label treatment with dupilumab with a first subcutaneous injection at 400 mg followed by 200 mg administration every 2 weeks, leading to both clinical, endoscopic and histological remission (13).

A recent Italian study by Visaggi et al. presented a case series evaluating dupilumab for induction and maintenance of remission in four adult patients with non-EoE EGIDs. The cohort included 2 patients with concurrent EoE and EoG; 1 patient with EoE, EoG and EoN and 1 patient presented EoE with both EoN and EoC. All patients underwent a standardized biopsy protocol over an 18-month follow-up period. Consistent with our study, the two adult patients with EoE and EoG were treated with dupilumab due to steroid-dependency, while the third patient for steroid resistance. All three achieved both clinical and histologic remission, without adverse events. The fourth patient (with EoE, EoN and EoC) who was treated for developing steroid-related adverse events, showed only a partial response to dupilumab: while histologic remission was achieved by week 16, endoscopy revealed a persistent ulcer of the ileocolonic anastomosis despite treatment. Furthermore, at the 16-month follow-up visit, the patient reported persistent symptoms of occasional bloating and diarrhea (25).

In our case, as in the above-mentioned Italian study (13), our patient was responsive to steroid treatments without experiencing side effects. However, in order to wean off steroids, we decided to administer dupilumab subcutaneously at a dosage of 300 mg once a week, as suggested for children with EoE in the manufacturer instructions, equally achieving symptoms remission and endoscopic and histologic normalization. Moreover, in our case, as in Lionetti's report (13), dupilumab has proved to be efficient in the treatment of both EoE and EoG in children. Differently from what previously described (11, 13), we demonstrated that dupilumab can be used in a single-therapy approach, without dietary limitations, in steroid-dependent children to avoid the development of steroid side effects. To our knowledge, this is the first case showing the efficacy of dupilumab in a child who suffers from non-EoE EGID but no other atopic conditions.

While the exact pathophysiologic mechanism underlying non-EoE EGID remains unclear, it appears to be related, like in other atopic conditions, to cell-mediated responses, including T-helper 2 cytokines (IL-4, IL-5, and IL-13) and chemokines (eotaxin1, eotaxin-3, and *α*4*β*7 integrin) (9, 10, 20, 26).

In fact, an italian study showed a higher prevalence of atopic comorbidity in patients with EoC compared with EoE, suggesting a shared epigenetic background between non-EoE EGID and Th-2 respiratory inflammation (27).

Dupilumab is currently approved in children older than 12 years to treat moderate to severe atopic dermatitis, asthma, chronic rhinosinusitis with nasal polyposis, and eosinophilic esophagitis. Although the clinical trial experimenting with dupilumab in patients aged 12-70 years with eosinophilic gastritis is ongoing (ClinicalTrials.gov number NCT03678545), and no studies in non-EoE EGID are available, the success of dupilumab treatment in the previously mentioned diseases, along with its action in blocking both the IL-4 and IL-13 cytokine cascade, make it a logical option for treating non-EoE EGID (28). Furthermore, the reduction in mucosal eosinophilia in our case supports the hypothesis that Th2 inflammation plays a key role in the pathogenesis of EoG, rendering dupilumab a viable therapeutic option for patients with EoG.

In any case, the choice of treatment should be thoroughly discussed with parents and patients, evaluating risks, benefits, and possible side effects.

In conclusion, our case presents a unique perspective on the successful use of dupilumab as a rescue therapy for a pediatric patient with concurrent EoE and EoG unresponsive to elimination diet. Despite initial responsiveness to steroid treatments, the patient faced challenges in tapering off oral steroids without relapse.

This case contributes to the growing body of evidence supporting the efficacy of dupilumab in treating EoE and extends its application to non-EoE EGIDs. Importantly, our patient, without any other atopic conditions, demonstrated positive outcomes with dupilumab therapy, highlighting its potential as a well-tolerated and effective treatment option.

As the medical community explores emerging treatments for EGIDs, the choice of therapy should be a collaborative decision, thoroughly discussed with parents and patients, considering risks, benefits, and potential side effects. Further research and clinical trials are warranted to broaden our understanding of the optimal use of dupilumab and other monoclonal antibodies in the management of pediatric EGIDs.

## Data Availability

The raw data supporting the conclusions of this article will be made available by the authors, without undue reservation.

## Supplementary material

Supplementary Material 1 Gastric biopsy of oxyntic mucosa showing mild superficial chronic infiltrate with occasional eosinophils [Hematoxylin-eosin 15x].

Supplementary Material 2 Esophageal biopsy showing normal architecture and occasional intraepithelial eosinophils (arrow) [Hematoxylin-eosin 15x].

Supplementary Material 3 Esophageal biopsy showing, basal hyperplasia, spongiosis and severe eosinophils intraepithelial infiltrate with eosinophilic microabscess (arrows) [Hematoxylin-eosin 25x].

Supplementary Material 4 Gastric biopsy of oxyntic mucosa after dupilumab therapy. Gastric biopsy showing superficial inflammatory infiltrate (hematoxylin-eosino 4X) (a). Gastric biopsy showing superficial lymphoplasmacellular infiltrate intermixed with some eosinophilic granulocytes (arrows). Fibrosis of superficial lamina propria it is not evident anymore. (hematoxylin-eosin 20X) (b).
