# Supplementary figures and images for: Dupilumab as a rescue therapy for steroid-dependent eosinophilic gastritis in a child unresponsive to elimination diet: a case report
Source: Front Pediatr. 2026 Apr 13;14:1755630. doi: 10.3389/fped.2026.1755630 (PMC13111380; doi:10.3389/fped.2026.1755630)

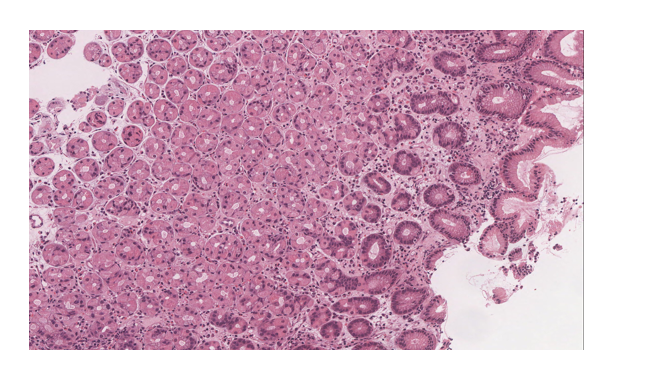

Supplement: Supplementary Material 1 — Gastric biopsy of oxyntic mucosa showing mild superficial chronic infiltrate with occasional eosinophils [Hematoxylin-eosin 15x]. [file Image1.jpeg]

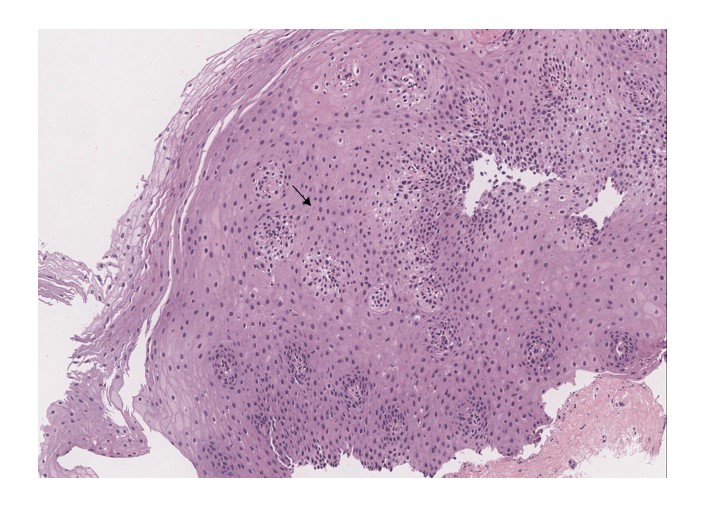

Supplement: Supplementary Material 2 — Esophageal biopsy showing normal architecture and occasional intraepithelial eosinophils (arrow) [Hematoxylin-eosin 15x]. [file Image2.jpeg]

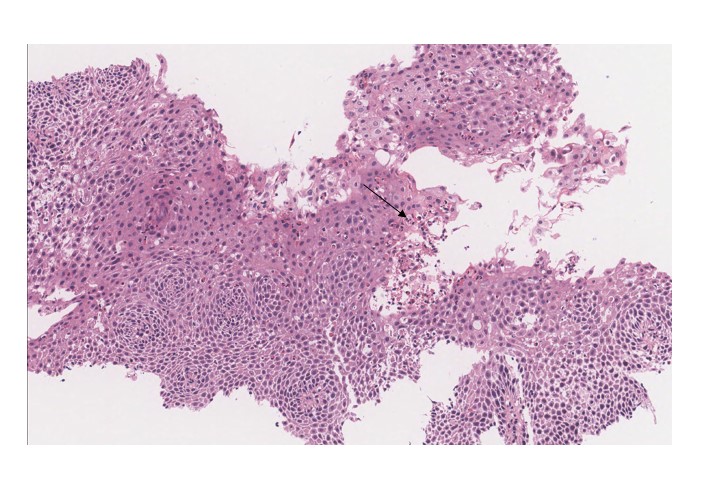

Supplement: Supplementary Material 3 — Esophageal biopsy showing, basal hyperplasia, spongiosis and severe eosinophils intraepithelial infiltrate with eosinophilic microabscess (arrows) [Hematoxylin-eosin 25x]. [file Image3.jpeg]

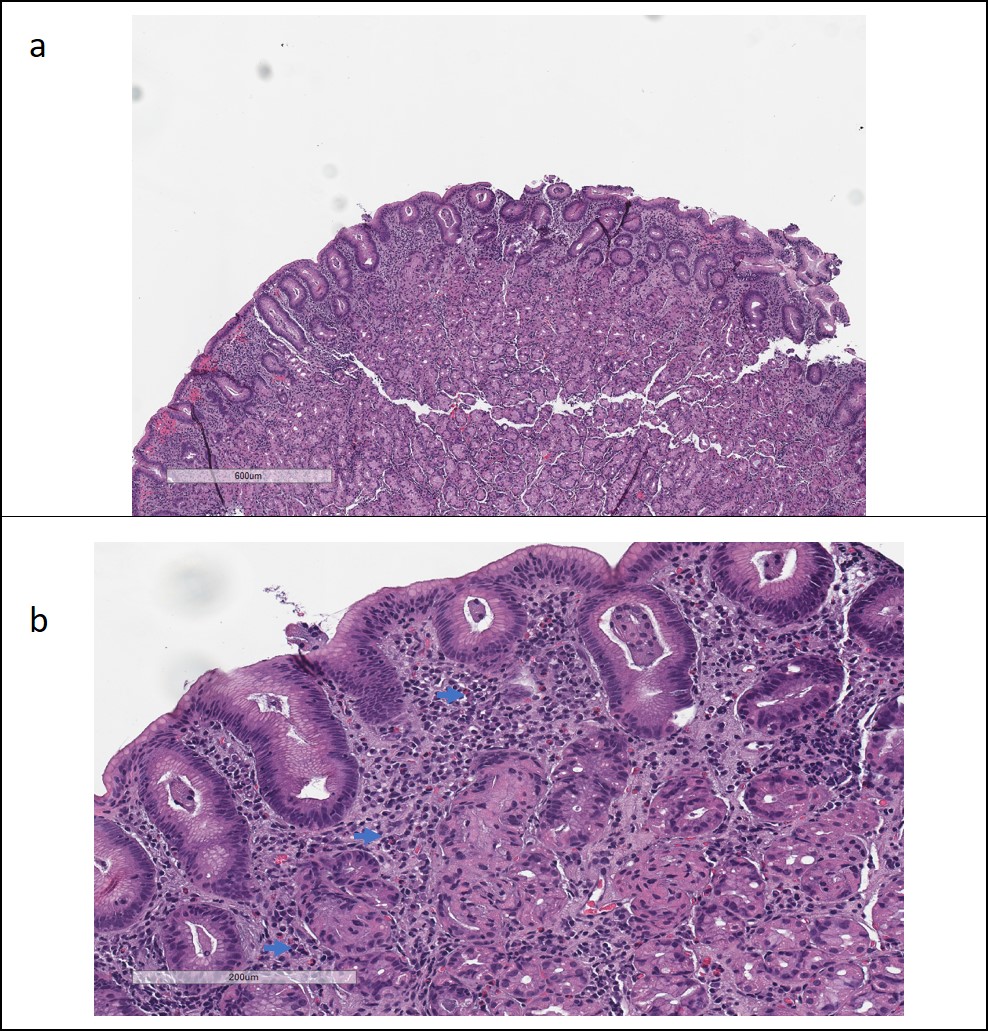

Supplement: Supplementary Material 4 — Gastric biopsy of oxyntic mucosa after dupilumab therapy. Gastric biopsy showing superficial inflammatory infiltrate (hematoxylin-eosino 4X) (a). Gastric biopsy showing superficial lymphoplasmacellular infiltrate intermixed with some eosinophilic granulocytes (arrows). Fibrosis of superficial lamina propria it is not evident anymore. (hematoxylin-eosin 20X) (b). [file Image4.jpeg]

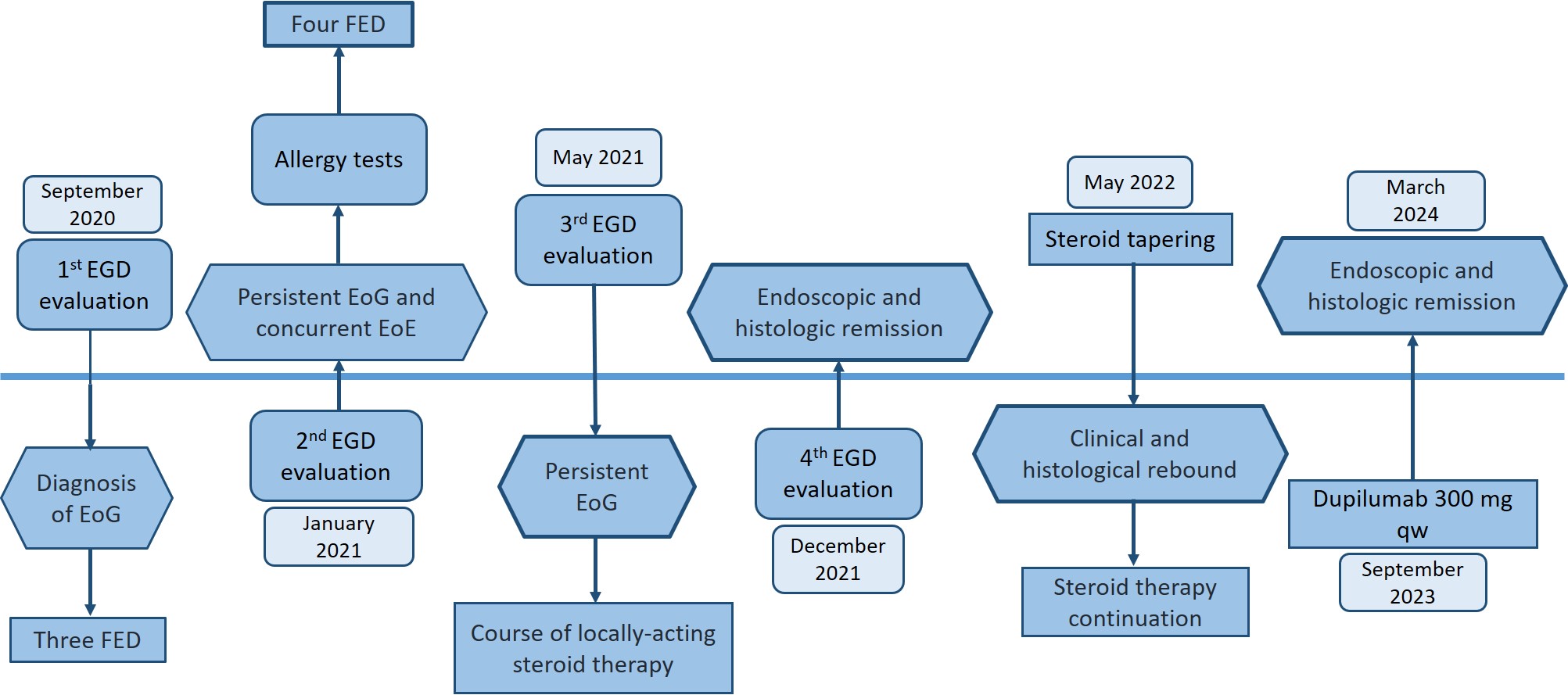

Supplement: Supplementary file 5 [file Image5.jpeg]
